# An international phase II trial and immune profiling of SBRT and atezolizumab in advanced pretreated colorectal cancer

**DOI:** 10.1186/s12943-024-01970-8

**Published:** 2024-03-23

**Authors:** Antonin Levy, Daphné Morel, Matthieu Texier, Roger Sun, Jerome Durand-Labrunie, Maria E Rodriguez-Ruiz, Severine Racadot, Stéphane Supiot, Nicolas Magné, Stacy Cyrille, Guillaume Louvel, Christophe Massard, Loic Verlingue, Fanny Bouquet, Alberto Bustillos, Lisa Bouarroudj, Clément Quevrin, Céline Clémenson, Michele Mondini, Lydia Meziani, Lambros Tselikas, Rastilav Bahleda, Antoine Hollebecque, Eric Deutsch

**Affiliations:** 1 Department of Radiation Oncology, Gustave Roussy, 114 Rue E. Vaillant, 94850 Villejuif, France; 2 INSERM U1030, Radiothérapie Moléculaire, Villejuif, France; 3 Faculty of Medicine, Université Paris Saclay, Le Kremlin-Bicêtre, France; 4 Biostatistics and Epidemiology Office, Gustave Roussy, Villejuif, France; 5 Oncostat 1018 INSERM, University Paris-Saclay, Villejuif, France; 6 Department of Radiation Oncology, Clínica Universidad de Navarrra, Pamplona, Spain; 7 Department of Radiation Oncology, Centre Léon Bérard, Lyon, France; 8 Department of Radiation Oncology, Institut de Cancérologie de L’Ouest-Centre Rene Gauducheau, St Herblain, Nantes, France; 9 Department of Radiation Oncology, Institut Bergonié, Bordeaux, France; 10 Drug Development Department (DITEP), Gustave Roussy-Cancer Campus, Villejuif, France; 11 Product Development Medical Affairs, F Hoffmann-La Roche Ltd, Basel, Switzerland; 12 Bioinformatic Platform, Gustave Roussy, Villejuif, France; 13 Department of Interventional Radiology, Gustave Roussy, Villejuif, France

## Abstract

**Background:**

Immuno-radiotherapy may improve outcomes for patients with advanced solid tumors, although optimized combination modalities remain unclear. Here, we report the colorectal (CRC) cohort analysis from the SABR-PDL1 trial that evaluated the PD-L1 inhibitor atezolizumab in combination with stereotactic body radiation therapy (SBRT) in advanced cancer patients.

**Methods:**

Eligible patients received atezolizumab 1200 mg every 3 weeks until progression or unmanageable toxicity, together with ablative SBRT delivered concurrently with the 2nd cycle (recommended dose of 45 Gy in 3 fractions, adapted upon normal tissue tolerance constraint). SBRT was delivered to at least one tumor site, with at least one additional measurable lesion being kept from the radiation field. The primary efficacy endpoint was one-year progression-free survival (PFS) rate from the start of atezolizumab. Sequential tumor biopsies were collected for deep multi-feature immune profiling.

**Results:**

Sixty pretreated (median of 2 prior lines) advanced CRC patients (38 men [63%]; median age, 59 years [range, 20–81 years]; 77% with liver metastases) were enrolled in five centers (France: *n* = 4, Spain: *n* = 1) from 11/2016 to 04/2019. All but one (98%) received atezolizumab and 54/60 (90%) received SBRT. The most frequently irradiated site was lung (*n* = 30/54; 56.3%). Treatment-related G3 (no G4-5) toxicity was observed in 3 (5%) patients. Median OS and PFS were respectively 8.4 [95%CI:5.9–11.6] and 1.4 months [95%CI:1.2–2.6], including five (9%) patients with PFS > 1 year (median time to progression: 19.2 months, including 2/5 MMR-proficient). Best overall responses consisted of stable disease (*n* = 38; 64%), partial (*n* = 3; 5%) and complete response (*n* = 1; 2%). Immune-centric multiplex IHC and RNAseq showed that SBRT redirected immune cells towards tumor lesions, even in the case of radio-induced lymphopenia. Baseline tumor PD-L1 and IRF1 nuclear expression (both in CD3 + T cells and in CD68 + cells) were higher in responding patients. Upregulation of genes that encode for proteins known to increase T and B cell trafficking to tumors (CCL19, CXCL9), migration (MACF1) and tumor cell killing (GZMB) correlated with responses.

**Conclusions:**

This study provides new data on the feasibility, efficacy, and immune context of tumors that may help identifying advanced CRC patients most likely to respond to immuno-radiotherapy.

**Trial registration:**

EudraCT N°: 2015–005464-42; Clinicaltrial.gov number: NCT02992912.

**Supplementary Information:**

The online version contains supplementary material available at 10.1186/s12943-024-01970-8.

## Introduction

Immune checkpoint blockers (ICB), such as anti-PD-1/PD-L1, have become a standard treatment in several tumor locations. The benefit of immunotherapy has been largely driven by a subset of patients experiencing durable tumor responses: overall, about 15% to 60% of patients respond to immunotherapy-based approaches, depending on tumor type, tumor mutational burden features (e.g., DNA mismatch repair-deficient [dMMR]/microsatellite instability-high [MSI-H]) and PD-L1 expression levels [1–3]. In several unselected tumor subtypes, such as in advanced colorectal cancer (CRC), studies evaluating ICB showed disappointing results, with median progression-free survival [PFS] and overall survival [OS] of 2.2 and 5.0 months, respectively, in a cohort with proficient MMR (pMMR) colorectal cancers treated with pembrolizumab [3]. Similarly, within the MSI-H/dMMR subpopulation of patients with CRC (which accounts for less than 5% of all advanced CRC), median PFS ranged from 2.3 to 4.1 months according to the number of prior lines [4].

An increasing number of preclinical [5–7] and clinical [8–11] data suggest that radiation therapy can enhance the immune anticancer response. Higher access to stereotactic body radiotherapy (SBRT) now allows to sharply target tumor lesions, reducing collateral damages to adjacent organs including lymph nodes [6], while inducing immunogenic cell death, which promotes a T-cell-mediated immune response against antigens derived from dying tumor cells. Ionizing radiations also enhance the expression of MHC-I molecules favoring antigen presentation and activate the interferon (IFN) cGAS-STING DNA-sensing pathway, contributing to the amplification of a tumor-directed adaptive immune response.

Conversely, radiation therapy may also promote immunosuppressive effects including lymphocyte exhaustion and subsequent PD-L1 upregulation, attraction of immunosuppressive cells (e.g.: myeloid cells and regulatory T cells), immunosuppressive cytokines release, and/or radiation-induced lymphopenia (RIL) [5–7]. RIL is frequent across all tumor types, often lasts several months after completion of radiotherapy, and has been shown to directly impact survival outcomes [6, 12, 13]. For now, the best way to use radiation therapy to enhance immunotherapy efficacy with limited radio-induced immunosuppressive effects remains unclear.

More clinical and deep irradiated tumor data are needed for optimizing the combination regimens and the selection of patients who would best benefit from immuno-radiotherapy combinations. In this single-arm phase 2 study, we assessed the safety, efficacy and biological correlate analysis of SBRT in combination with the PD-L1 inhibitor atezolizumab in advanced pretreated cancer patients. Here, we report the results of the CRC cohort.

## Patients and methods

### Study design and participants

In this international, multi-center single-arm phase 2 trial, we enrolled different cohorts of patients including advanced CRC, renal cell carcinoma, non-small cell lung cancer (NSCLC) and sarcomas who had at least 1) one lesion eligible for SBRT and 2) one unirradiated lesion, both measurable by Response Evaluation Criteria in Solid Tumors, version 1.1 (RECIST v1.1). Additional key eligibility requirements included Eastern Cooperative Oncology Group Performance Status (PS) of 0 to 1, absence of autoimmune or immunodeficiency diseases, and adequate organ function. CRC patients should have been considered in treatment failure as per the current standard recommendation. We placed no limit on the number of prior therapies, although patients were not eligible if they had received prior PD-1 or PD-L1 inhibitor. Patients were eligible regardless of their PD-L1 or molecular target/MSI status. The trial was approved by the relevant ethics/institutional review board and was completed in accordance with international standards of good clinical practice. All patients provided written informed consent at the time of enrolment.

### Procedures

Intravenous atezolizumab therapy, 1200 mg, was administered every 21 days (3 weeks). Hypofractionated SBRT was delivered concurrently with the 2nd cycle (week 6, Figure S1) at an ablative dose, using 6MV photons with standard field encompassing tumor. SBRT was delivered at a recommended dose of 45 Gy in three fractions of 15 Gy (equivalent biologic dose (BED) > 100 Gy). The protocol allowed adapted doses based upon normal tissue tolerance constraints. The radiation dose was prescribed to the 90% isodose line in order to deliver 95% of the planned dose to 95% of the planned tumor volume (PTV). SBRT was applied to at least one tumor, and another untreated tumor site was required to be evaluable by RECIST 1.1. Central thoracic and brain lesions were not eligible for SBRT in this study but they could be considered as “not treated” evaluable metastases. Treatment with atezolizumab continued for up to two years in the absence of documented disease progression, unacceptable adverse events, intercurrent illness precluding further administration of treatment, the investigator’s decision to withdraw the participant, participant withdrawal of consent, pregnancy of the participant, or non-adherence with trial treatment.

### Immune profiling

After having signed a dedicated informed consent for translational research purposes, patients underwent sequential tumor biopsies of the irradiated lesion at baseline, week 3 (pre-SBRT) and week 7 (post-SBRT) for biomarker analysis. In some cases, lesions strictly outside of the radiation field were also biopsied at the week 3 and week 7 timepoints to study potential abscopal impacts of SBRT. Tumor samples were both formalin fixed paraffin embedded (FFPE) and freshly frozen, respectively for immunohistochemistry (IHC) analysis and whole RNA extraction and sequencing. For each sample, tumor cellularity was assessed by a senior pathologist on a haematoxylin–eosin-saffron (HES)-stained slide from the FFPE-preserved biopsy. Samples with no tumor cells were excluded from the analyses.

FFPE blocs were sliced into 4-μm large sections to perform IHC multiplexing. An immune-targeting panel was developed at the experimental and translational pathology (PETRA) platform of Gustave Roussy according to the following (chromogenic library): 2Plex CD163/CD68 (anti-CD163 ref Mob460-05 clone 10D6 from DBS; anti-CD68 ref M0876 clone PG-M1 from DAKO), 4Plex CD8/PD-L1/FoxP3/cytokeratin (CK) (anti-CD8 ref 05937248001 clone SP57 from Roche; anti-PD-L1 ref 7994190001 clone SP263 from Roche; anti-FoxP3 ref ab99963 clone SP97 from Spring; anti-CK ref Mob190.05 clone AE1-AE3 from DBS) and 4Plex IRF1/CD20/CD3/CD68 (anti-IRF1 ref 8478 clone D5E4 from Cell Signaling; anti-CD20 ref M075501-2 clone L26 from DAKO; anti-CD3 ref A0452 polyclonal from DAKO; anti-CD68 ref M0876 clone PG-M1 from DAKO). Once stained, slides were digitalized at 20X using a VS120 scanner (Olympus Life Science). Finally, cellular densities of label-positive cells were automatically assessed using the HALO® image analysis software, in-situ hybridization module.

Tumor whole RNA was extracted retrospectively by batch using the AllPrep RNA Mini Kit (Qiagen) following the manufacturer’s instruction. RNA extracts were sent to Novogene to perform human mRNA sequencing after rRNA removal on an Illumina NovaSeq 6000 system, PE150, Q30 ≥ 85%. Raw data recorded as FASTQ files were processed by the bioinformatics platform of Gustave Roussy including quality control, preprocessing, aggregation/normalization steps and differential expression analyses. Unsupervised hierarchical clustering was performed on the log2-transformed TPM value differences between week 3 (pre-SBRT) and baseline samples considering 536 immune-related genes among the previously described LM22 gene set [14]. Euclidean distances were used. Immune deconvolution from bulk RNA sequencing data was performed using the CIBERSORTx tool [15]; “abs” option was used to obtain absolute scores compatible with inter-sample comparison.

### Objectives and outcome measures

The primary efficacy endpoint was the one-year PFS rate. PFS was defined from the start of atezolizumab treatment to the first documented disease progression, death due to any cause. Response status was based on investigator assessment of scans using RECIST 1.1. Scans of all previously involved disease sites were performed at week 4, 7, 13, and then every 12 weeks or as clinically indicated. Secondary end-points included safety, as defined by Common Terminology Criteria for Adverse Events (CTCAE v4.03) toxicity profile, overall survival (OS), and objective response rates (ORRs). Patients who had a PFS of more than one year were described as “elite responders”.

### Statistical analyses

Each cohort was analyzed separately. In each cohort, a Fleming 1-stage design was applied to demonstrate that the PFS rate at one year is not inferior to 15% but could reach 32%. To test in each cohort, the hypothesis that the PFS rate is greater than p0 = 15% with alpha = 0.033 and with a 90% power to detect activity greater than p1 = 32%, 54 evaluable patients should be enrolled. If 13 patients or more are alive and free of progression at one year in the cohort, the combined SBRT + atezolizumab is considered a success for the corresponding histology. To account for possibly 10% of non-evaluable patients, 60 patients were enrolled per cohort. *P* ≤ 0.05 was considered statistically significant. Statistical analyses were performed using SAS software, version 9.4 or R software, version 3.0.2 (R Foundation for Statistical Computing).

Translational data were analyzed using Prism V.9 (GraphPad, California, USA). Unless otherwise stated, multigroup comparisons were done according to an ordinary one-way ANOVA calculation followed by Tukey’s multiple comparisons test. For simple comparison analyses, an unpaired Student’s t-test with Welch’s correction was used to compare data when assuming Gaussian distributions with unequal standard deviation across groups, while the Mann–Whitney test was used for non-parametric testing.

## Results

### Patients

**Fig. 1:**
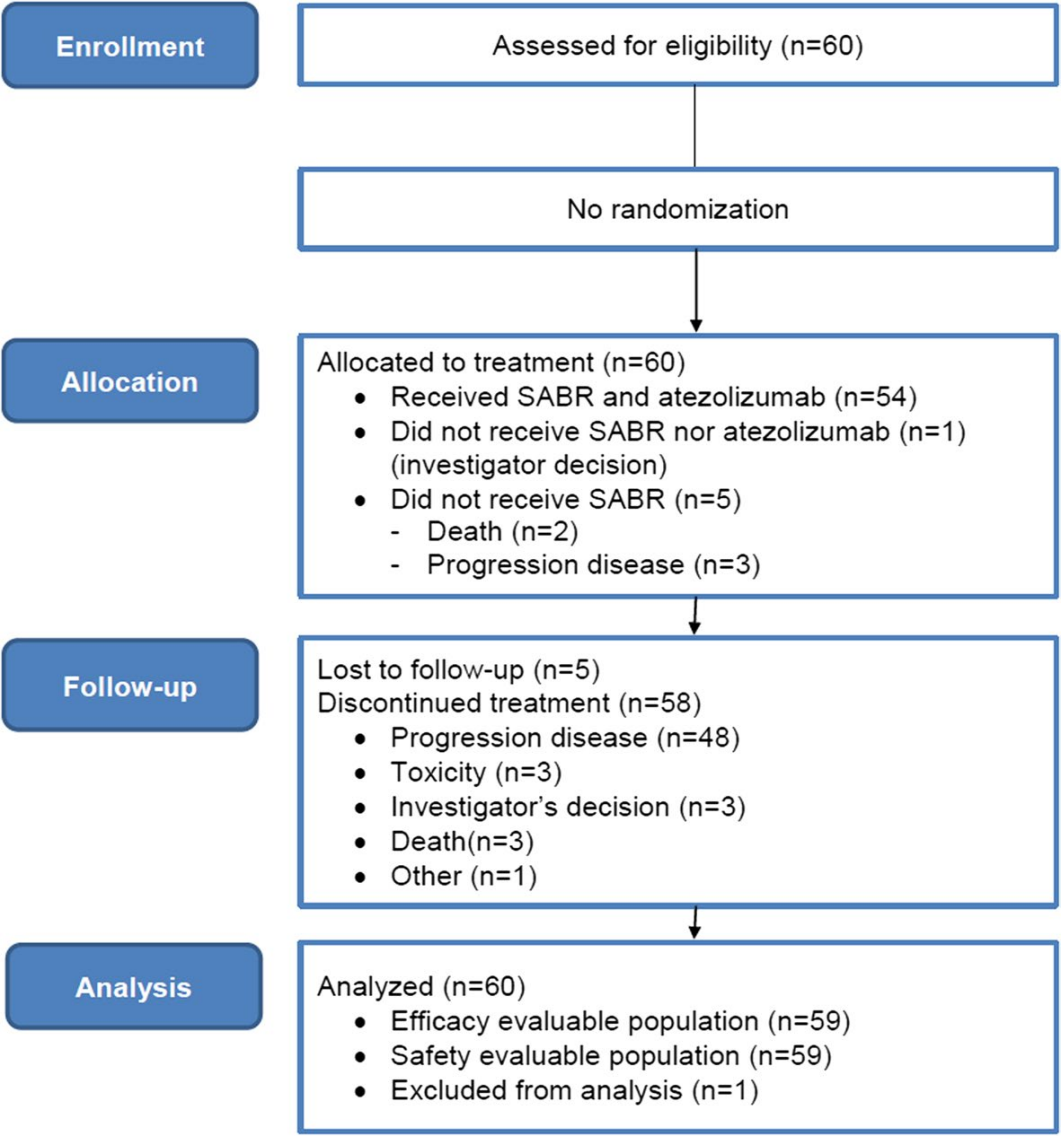
CONSORT Diagram

From November 2016 through April 2019, 60 eligible patients provided informed consent (Fig. 1) for trial entry, in five European centers (France: *n* = 4, Spain: *n* = 1). Most patients were male (38 men [63%] and 22 women [37%]), with a median age of 59 years [range, 20–81 years]). Of these, 59/60 underwent treatment with atezolizumab and 54/60 received SBRT. The Table 1 gives the baseline clinical characteristics of participants. Most patients (*n* = 46/60 [77%]) had liver metastases. Because some patients received multiple types of chemotherapy (median number of prior systemic treatment: 2, range 1–6) and surgery, the sum of subgroups exceeds the total of each modality. Thirty-two patients had adequate tissue for assessment of PD-L1 expression, and 29 had adequate tissue for assessment of CD8 T-cell infiltration. In patients undergoing testing, 11 (34%) had results positive for PD-L1 (≥ 1%) and 15 (52%) had CD8 T-cell infiltration of greater than 2.5%. A total of 8/41 (20%) patients with available status were MSI-H and molecular alterations were retrieved in 34/60 (57%) patients. One patient did not receive atezolizumab given investigator's decision (Bilirubin > 1,5N). In 59/60 treated patients, a median of 4 cycles of atezolizumab was delivered (range, 1–94). SBRT was delivered at a median dose of 45 Gy (range 21–45 Gy; with a median dose of 48.2 Gy covering 95% of PTV [20.9–54.8 Gy]) in 3 fractions (54/54, 100%) for a median duration of 4 days (3–8 days). The median PTV volume was 49.5 cc (6.3–325 cc). Main irradiated sites were lung (*n* = 30/54; 56.3%), liver (*n* = 18/54; 33.3%) or other locations (*n* = 8/54; 13.3%).

**Table 1:** Baseline clinical characteristics

**Variable**		**Total**	
		N	%
**Age at inclusion (years)**	Median (range)	59 (20–81)	
**Gender**	Male	38	63
	Female	22	37
**ECOG PS**	0	28	47
	1	32	53
**T stage**	T2	3	6
	T3	24	45
	T4a	16	30
	T4b	6	11
	Tx	4	8
**N stage**	N0	11	21
	N1	17	33
	N2a	15	29
	N2b	4	8
	Nx	5	10
**Metastatic sites**^**b**^	Liver	46	77
	Lung	45	52
	Peritoneal cavity	14	23
	Others	10	17
**Number of metastatic sites**	1	10	17
	2	27	45
	3	16	27
	> 3	7	17
**Prior treatments**	Surgery	49	82
	Radiotherapy	19	68
	Chemotherapy + TT	58	97
	Median N lines (range)	2 (1–5)	
**Molecular status**	PDL1 ≥ 1%	11	18
	MSS NA	19	32
	pMMR	33	55
	MSI-H	8^a^	13
	KRAS/NRAS	25	42
	HER2	3	5
	MET	1	2
	PI3KCA	1	2
	BRAF	6	10
**Atezolizumab**	median N cycles (range)	4 (1–94)	
**SBRT (Gy)**	Median dose (range)	45 (21–45)	
**Irradiated sites**	Lung	30	56
	Liver	18	33
	Others	8	13

^a^One did not receive atezolizumab

^b^Patients had several metastatic sites so sum of percentages is above 100

### Efficacy

**Fig. 2:**
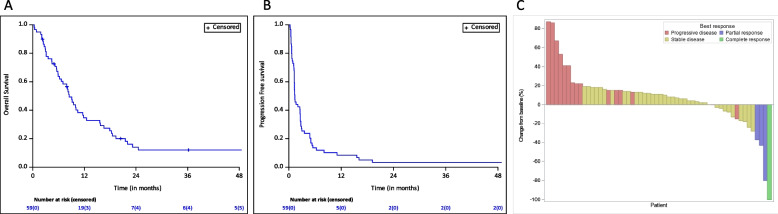
Clinical outcomes. **A**-**B** Kaplan Meyer describing progression-free (**A**) and overall survival (**B**) in the intention-to-treat population. **C** Best change from baseline in size of target lesions in evaluable patients

The database was locked on 13/04/2023 and 59 patients were included in the efficacy analysis. After a median follow-up of 8.3 months (95% CI: 5.9–10 months), 5 patients were alive and 2 had no evidence of disease. The median OS was 8.4 months (95% CI: 5.9–11.6 months, Fig. 2A). The one-year PFS rate and median PFS were 8.5% [95% CI: 3.7—18.4] and 1.4 months [95% CI: 1.4- 2.6], respectively (Fig. 2B). MSI-H (vs pMMR) was associated with higher median PFS (6.3 vs 1.3 months; *p* = 0.02), although median OS were not significantly different (11.8 vs 8.3 months, *p* = 0.08; Figure S2). Five “elite” patients (e.g. with on-treatment PFS ≥ 1 year) had their disease controlled more than one year after treatment start (median time to progression among elite responders: 19.2 months (range 15.3–60.6 months), with 2/5 pMMR and 3/5 MSI-H); with 2 patients still pursuing atezolizumab at last news. There was no observable objective response at 7 weeks, although stable disease [SD] was observed in 23/59 (39%) patients. Best overall responses were: SD (*n* = 38; 64%) and four patients had an objective response (partial response [PR], *n* = 3; complete response [CR], *n* = 1) (Fig. 2C). Among elite patients 2/5 had objective responses (PR, *n* = 1; CR, *n* = 1).

### Safety

Atezolizumab and SBRT combination was safe, with no new toxicity signals. There was no increase of infield toxicity and no increase of incidence or pattern of immune-related adverse events (AEs) (Table 2). Out of the 59 evaluable patients, 30 (51%) experienced severe (grade 3–5) AEs (Table S1). Overall, 26 patients (44%) experienced serious AEs, including 18 (30%) grade 3–5. No grade 4 or 5 attributable to treatments adverse events occurred. Grade 2 atezolizumab-related AEs included: autoimmune hepatitis (*n* = 1; 2%), hepatic cytolysis (*n* = 1; 2%) and myositis (*n* = 1; 2%). No SBRT-related grade ≥ 3 toxicity was reported (Table 2).

**Table 2:** Treatment-related (A: atezolizumab and B: SBRT) Adverse Events in Study Population per CTCAE

**A**				
		Grade		
**SOC**	Preferred term	1	2	3
**Blood and lymphatic system disorders**	Lymphopenia		1 (2%)	
**Gastrointestinal disorders**	Dysphagia	1 (2%)		
**General disorders and administration site conditions**	Chills	1 (2%)		
**Hepatobiliary disorders**	Hepatic Cytolysis			1 (2%)
**Immune system disorders**	Autoimmune Hepatitis			1 (2%)
**Investigations**	CPK increased		1 (2%)	
	Weight loss	1 (2%)		
**Metabolism and nutrition disorders**	Anorexia	1 (2%)		
**Musculoskeletal and connective tissue disorders**	Myalgia	1 (2%)		
	Myositis			1 (2%)
**Respiratory, thoracic and mediastinal disorders**	Dyspnea	1 (2%)		
	Pneumonitis		1 (2%)	
**Skin and subcutaneous tissue disorders**	Folliculitis	1 (2%)		
	Psoriasis	1 (2%)		
**B**				
		Grade		
**SOC**	Preferred term	1	2	
**Infections and infestations**	Urinary tract infection		1 (2%)	
**Neoplasms benign, malignant and unspecified**	Tumor pain	1 (2%)	1 (2%)	

### Responders display enriched immune profiling

We performed exploratory analyses to determine whether pathological features could be associated with clinical outcomes. Among the 59 evaluable patients, 12 (20%) underwent tumor biopsy(ies) for research purposes, allowing the retrieval of 27 samples: 11 were collected at baseline, 10 at week 3 (pre-SBRT) and 6 at week 7 (post-SBRT). Most (7/12; 58%) biopsied sites were liver metastases and all were irradiated or were planned to be irradiated (“in-field” lesions). Biopsies collected in out-of-the-field locations were not considered in this analysis because of their limited number. Details are provided in Table S2. Complete blood counts were also extracted for those patients. Patients were divided into two groups according to their response profile after 12 weeks of treatment as per RECIST v1.1 criteria: patients who remained stable or who showed tumor response (including “elite” responders) were considered “SD/PR/CR” while patients who had progressed were considered “PD”.

**Fig. 3:**
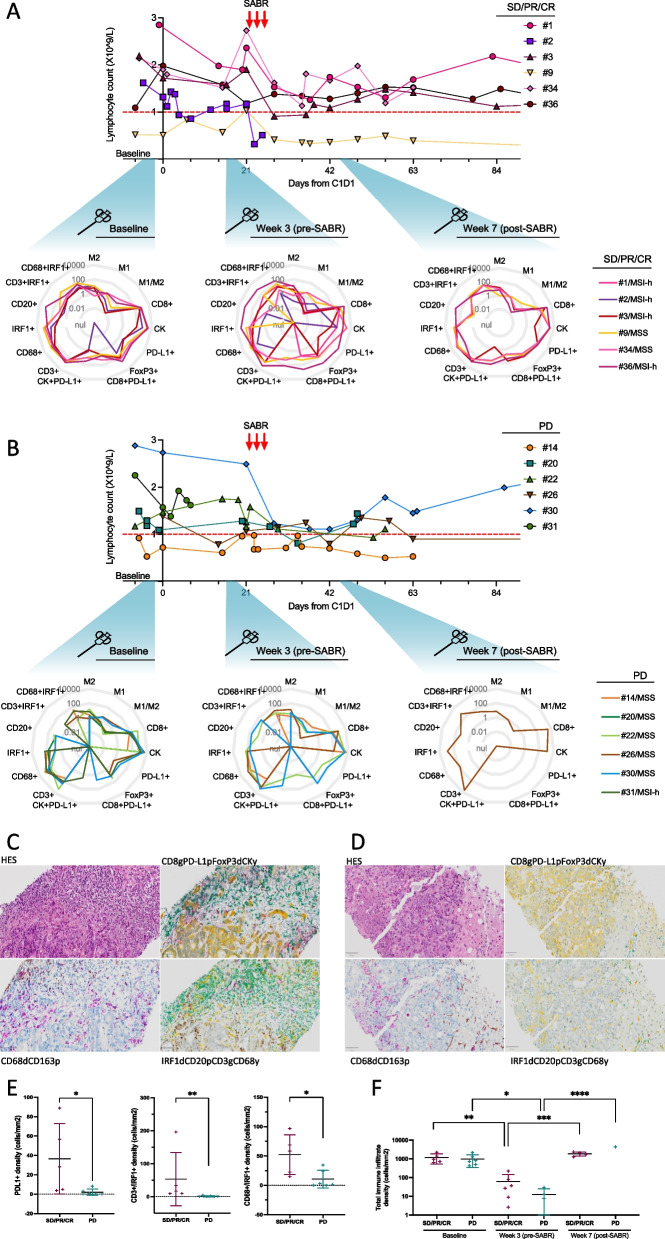
Biopsies immune profiles and lymphocyte count. **A**-**B** (Top panels). Absolute lymphocyte blood counts from baseline evaluation to the fifth cycle of atezolizumab (D84) in patients who experienced stable disease, partial response or complete response as best tumor response according to RECIST 1.1 (SD/PR/CR) (**A**) and in patients who rapidly progressed (PD) (**B**). Red arrows indicate SABR delivery during the course of treatment. The three periods of tumor biopsies are also indicated. Yellow and red stars refer respectively to MSI-high patients and elite responders (PFS > 1 year). (Bottom panels). Immunograms detailing the immune composition of the immune infiltrate as assessed by multiplex IHC in FFPE tumor biopsy samples collected at the indicated timepoints in patients with SD/PR/CR (**A**) an in patients who progressed (**B**). Each absolute cell density is ranged from 0 to 10.000 cells/mm2. **C**-**F** Multiplex IHC. Images at 20X of two liver samples with a high level (**C**) and a low level (**D**) of immune infiltration; (**E**); Comparison of tumor immune infiltration according to disease control rate (defined as lack of disease progression) in baseline biopsies by different IHC markers; (**F**) Scatter dot plot showing the sum of densities of immune infiltrating cells assessed by multiplex IHC in tumor samples and calcutated as follows: (macrophages density + CD3 + cells density + CD68 + density + CD20 + density). Mean with SD are represented. ns > 0.05; 0.05 ≤ * < 0.01; 0.01 ≤ ** < 0.001; 0.001 ≤ *** < 0.0001; 0.0001 ≤ ****. HES: Haematoxylin–Eosin-Saffran; d: DAB brown; p: purple; y: yellow; g: green

In our cohort, the kinetics of blood lymphocyte counts were similar in the two groups (Fig. 3A, B and S3). There was a trend towards lymphocyte count reduction upon SBRT treatment that was more rapidly transient in SD/PR/CR patients than in PD patients (mean change from pre-SBRT to D42: -28.5% in SD/PR/CR and -54.1% in PD patients, with median lymphocyte count at D42: 1.28X10^9/L in SD/PR/CR vs 0.79 X10^9/L in PD patients, *P* = 0.4; Figure S3A). Similarly, the neutrophil-to-lymphocyte ratios (NLR) were not different between groups (baseline NLR in responder vs PD patients: 5.32 vs 5.23, respectively; week 3 NLR: 4.06 vs 4.15; week 7 NLR: 6.99 vs 6.64, all *P* > 0.8; Figure S3B).

We observed variations in tumor immune infiltrate profiles as assessed by IHC multiplex analysis (Fig. 3C: responding patient, 3D). Baseline PD-L1 was more expressed on the cell surface of tumor cells of responding patients versus PD patients (cytokeratin + /PDL1 + co-labelling: median density 8.3 versus 0 positive cells/mm^2^, respectively, *P* value = 0.0087). In addition, IRF1 nuclear expression was higher in SD/PR/CR patients at baseline in CD3 + T cells (median density 16.7 versus 0 positive cells/mm^2^ in PD patients, *P* value = 0.0043) and in CD68 + cells (median density 58.4 versus 2.3 positive cells/mm^2^ in PD patients, *P* value = 0.0498) (Fig. 3E, Figure S4). We observed no differences between patient groups at week 3 samples, and no statistical analysis could be made at week 7 samples because only one sample was available in the PD group.

To assess the effect of treatment on the overall immune infiltrate, we roughly estimated the presence within the tumor of T and B lymphocytes and macrophages by computing the sum of CD3 + , CD20 + and CD68 + immunolabelled cells. We found that week 3 samples (pre-SBRT) comprised significantly fewer immune cells than baseline samples in both groups (*P* value = 0.0086 in SD/PR/CR and 0.0443 in PD patients, Fig. 3F), whereas the immune infiltrate within irradiated tumors increased substantially after SBRT (*P* value = 0.0001, in SD/PR/CR patients).

### Immune-related genes variation correlate with response

**Fig. 4:**
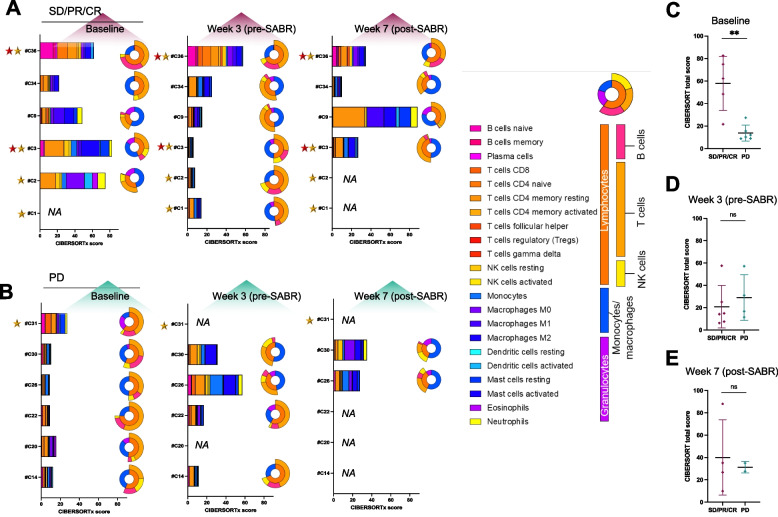
RNAseq immune cell differences in patient biopsies. **A**-**B** CIBESORTx characterization of the immune infiltrate obtained from RNAseq analyses performed on fresh frozen tumor samples in patients with stable disease, partial or complete response (SD/PR/CR) (**A**) and in patients who progressed (PD) (**B**), according to the treatment timepoint (baseline, week 3 and week 7). For each analyzed tumor sample, a bar chart depicts the absolute score of each immune subpopulation and is accompagned with a circular representation that indicates the relative fraction of granulocytes, monocytes/macrophages and lymphocytes within the sample, as indicated. Yellow and red stars refer respectivelly to MSI-high patients and elite responders (> 1 year). **C-E**. Scatter dot plots comparating the total CIBESORTx score found in patients with SD/PR/CR versus patients who progressed in tumor samples collected at baseline (**C**), at week 3 (**D**) and at week 7 (**E**). Mean with SD are represented. ns > 0.05; 0.01 ≤ ** < 0.001

We then used RNA sequencing (RNAseq) to estimate the abundances of the main immune cell subtypes using the CIBERSORTx deconvolution method. For each cell subset, the tool outputs an absolute score that reflects its representation within the bulk sample (Fig. 4A, B). Similarly to what was observed with IHC data, CIBERSORTx total scores were overall higher in SD/PR/CR patients than in patients with PD at baseline (mean CIBERSORTs total score: 58.0 in SD/PR/CR patients, 13.9 in PD patients; *P* value = 0.0087; Fig. 4C), suggesting that the tumor microenvironments of responding patients were more enriched with immune cells at baseline according to RNAseq data, although we found no specific immune feature that correlated with treatment response. No difference was found at W3 and W7 (Fig. 4D, E), possibly due to the small number of samples analyzed.

**Fig. 5:**
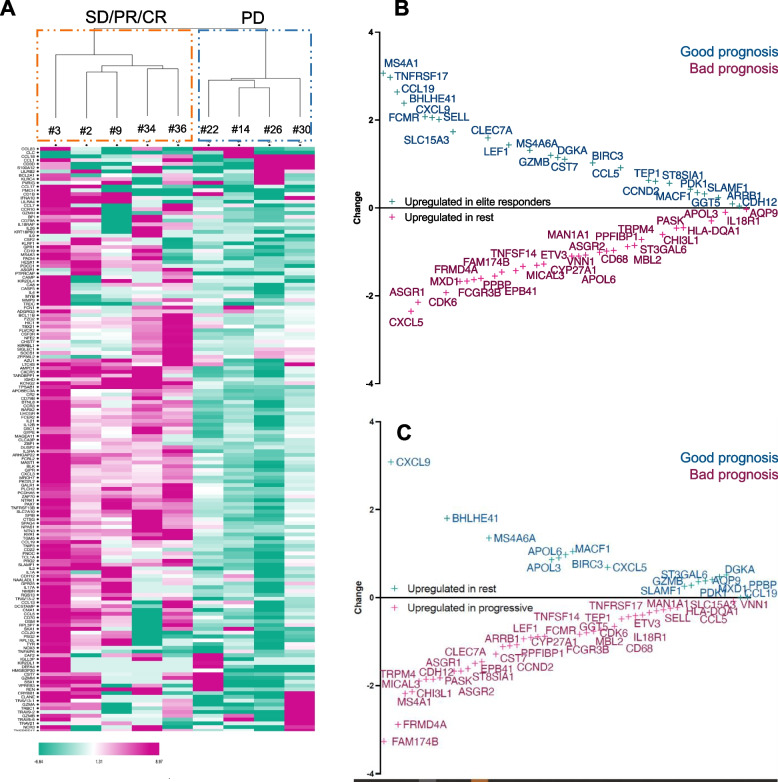
Coding gene RNA expression. **A** Heatmap showing an unsupervised hierarchical clustering (Euclidean distances) of the differences in immune gene expressions between before and after (week 3) treatment with atezolizumab. The clustering was made on log2-transformed TPM values of 536 immune-related genes extracted from the LM22 dataset after pre-processing, in patients for whom both baseline and week 3 RNAseq data were available. Pink refers to higher expression at baseline (reduction of expression at week 3) and green refers to lower expression at baseline (increased expression at week 3) **B**-**C**. Differential expression analyses of top-differentiated genes among the 536 immune-related genes when comparing only elite responders (> 1 year) with the others (**B**) and patients who rapidly progressed with the others (**C**). CCL19, BHLHE41, CXCL9, MS4A6A, GZMB, DGKA, BIRC3, PDK1, MACF1 and SLAMF1 expressions are commonly found of good prognosis

When performing an unsupervised hierarchical clustering of the most differently expressed immune genes (selected among the LM22 gene set signature) between week 3 and baseline samples, we observed that SD/PR/CR patients clustered altogether and were characterized by a decreased expression of most immune-related genes at week 3 compared to baseline, while this subset of genes was rather upregulated in PD patients (Fig. 5A). This suggests that atezolizumab may rapidly (by cycle 2) modify the immune features within the tumor microenvironment in non-responding patients towards a suboptimal state, which is consistent with the observation that patients who do not respond to anti-PD1/PDL1 treatment usually progress within a few weeks. To find out which immune genes were the most differentially expressed according to treatment response, we computed two differential expression analyses of all available samples taking into account the biopsy time point as a confounding factor. First, we compared RNAseq data from tumors of “elite” responders *versus* “non-elite” responders (Fig. 5B). The second analysis consisted in comparing immune expression profiles of all SD/PR/CR patients *versus* PD patients (Fig. 5C). The pooled analysis of genes that had significantly different expression levels between responders (either “elite” or all SD/PR/CR) and non-responders from this immune gene cluster revealed increases in genes that encode for proteins known to increase T and B cell trafficking to tumors (CCL19, CXCL9), migration (MACF1) and tumor cell killing (GZMB).

## Discussion

In this international single-arm phase II trial, we assessed the safety and the efficacy of SBRT combined with atezolizumab in unselected advanced CRC. Treatment was well tolerated with no unexpected toxicity (no grade 3 or more SBRT-related AE). The median PFS was modest (1.4 months [95% CI: 1.4- 2.6]; 6.3 months in MSI-H patients) in this unselected and pretreated population, as previously reported [3, 4]. However, five (8%) “elite” patients had their disease controlled for longer than one year, two of whom were pMMR, which suggests that SBRT might have boosted the efficacy of atezolizumab in those patients.

Single or double agent ICB is considered an ineffective approach for pMMR CRC, with median PFS not exceeding 2.5 months in previous reports [3, 16]. Presumed mechanisms include the low antigenicity [17] and the high immunosuppression of those tumors, with a possible influence of liver metastases that have been shown to affect the overall ICB efficacy. For example, in a retrospective study performed on 95 patients with pre-treated pMMR CRC receiving an anti-PD1/PDL1 therapy, the overall response rate was of 19.5% in patients without liver metastases, and no response (0%) was observed among the 54 patients with liver metastases [18]. The median PFS was also significantly higher in patients without liver lesions (4.0 months vs 1.5 months, respectively for patients without and with liver metastases). Patients with hepatic lesions may also experience an impaired tumor infiltration with (cytotoxic) T-cells, a phenomenon also called hepatic syphoning [19]. One hypothesis underlying this observation is that the liver microenvironment could be directly immunosuppressive through the increase of Treg and myeloid-derived suppressive cells (MDSC) [20] and subsequently, triggering the depletion of cytotoxic CD8^+^ T and NK cells [21]. Considering this, liver-directed ablative radiotherapy could represent a promising option to relaunch a systemic antitumor immunity in patients receiving ICB [21].

Our study provides new data on the tumor infiltrate characterization and its dynamic throughout immuno-radiotherapy treatment, which may help selecting advanced CRC patients most likely to respond to immuno-radiotherapy regimens. First of all, immune-centric multiplex IHC and RNAseq suggested that SBRT redirected immune cells towards tumor lesions, even in the case of RIL (Fig. 3). This is consistent with what was observed in another phase II study in which patients with pretreated p-MMR advanced CRC received durvalumab-tremelimumab and radiotherapy. In this study, median PFS was of 1.8 months [95% CI 1.7–1.9 months], *n* = 21/24 included patients had flow cytometry on peripheral blood mononuclear cells (PBMC) and authors could observe that CD8 + T lymphocytes were activated only among the two responders [22]. In addition, in our analysis, we could notice that the change of RNAseq expression of immune-related genes between before and after the start of atezolizumab occurred in opposite directions according to the response group (Fig. 5A). This suggests that primarily-resistant immune systems may be identified as soon as the second cycle of atezolizumab. Importantly, T-cell interferon activation and increased expression of chemotaxis signals such as CXCL9 were observed in responding patients. Similar findings were shown in a SBRT-pembrolizumab prospective cohort of 68 patients with advanced tumors. The same work reported that elevated expression of *TGFb* correlated with less tumor responses [23]*.* Other studies showed that radiation therapy and TGFβ inhibition could increase immune infiltration [24] and clinical response [25]. Interestingly, a recent trial assessing the efficacy of the bispecific antibody bintrafusp alfa (TGFβ-trap and anti-PD-L1) in patients with liver-limited MMRp mCRC was stopped early for loss of equipoise after 4 enrolled patients (out of 15 planned) [26]. This suggests that the presumed deleterious impact of liver metastases may not be counteracted by the dual blockade of TGFβ and PD-L1.

This study has limitations. The true effect of SBRT is difficult to assess given the single arm design. The trial started in 2016 and the type of irradiation used (concomitant high dose-fractionation mostly delivered to a single site) might not be the more immunogenic regimen. Although debated [27, 28], recent reports suggest that multisite irradiation delivered at a lower dose (e.g. 3 × 8 Gy) before ICB initiation could be more effective [5–7]. Some other biomarkers such as circulating tumor DNA [26], immunoscore [29], aneuploidy [30] or tumor mutational burden (TMB) were not assessed. In a phase II trial including 40 patients with MSS CRC, Parikh et al. did not find differences of TMB according to response after the combination of radiation therapy, ipilimumab and nivolumab [31]. We could not integrate out of field tumor heterogeneity in the analysis due to the small size of most tumor biopsy samples that we retrieved. Alternatively, this could have been guided by imaging feature analysis using artificial intelligence-guided methods such as radiomics [32], although this would have required a larger sample size to provide more tangible information. By comparison, a study in patients with HPV-unrelated head and neck squamous cell carcinomas described the high-dimensional multi-omics and spatial data analysis assessed on surgical specimens (*n* = 21) in patients treated in a neoadjuvant phase I/II trial of SBRT with single-dose anti-PD-L1 durvalumab [33]. Responders displayed an increase in post-treatment effector T cells and antigen presentation**.**

## Conclusion

This study suggests that SBRT induces beneficious stromal changes and can modify the clinical pattern of intrinsic resistance in patients with advanced pretreated CRC who are candidates to ICB therapy. We identified several tumor-related immune features that correlated with treatment outcome in our cohort, such as baseline tumor PD-L1 and IRF1 nuclear expression, which could, if validated, help guiding ultraprecision radiation therapy combination with ICB.

### Supplementary Information

**Supplementary Material 1.**

## Data Availability

Not applicable.

## Abbreviations

ICB: Immune checkpoint blockers
dMMR: Mismatch repair-deficient
MSI-H: Microsatellite instability-high
PFS: Progression free survival
OS: Overall survival
pMMR: Proficient MMR
SBRT: Stereotactic body radiotherapy
IFN: Interferon
RIL: Radiation-induced lymphopenia
NSCLC: Non-small cell lung cancer
PS: Performance Status
BED: Euivalent biologic dose
PTV: Planned tumor volume
IHC: Immunohistochemistry
HES: Haematoxylin-eosin-saffron
SD: Stable disease
PR: Partial response
CR: Complete response
NLR: Neutrophil-to-lymphocyte ratios
RNAseq: RNA sequencing (RNAseq)

## References

[1] Herbst RS, Giaccone G, de Marinis F, Reinmuth N, Vergnenegre A, Barrios CH (2020). Atezolizumab for First-Line Treatment of PD-L1-Selected Patients with NSCLC. N Engl J Med.

[2] Robert C, Ribas A, Schachter J, Arance A, Grob JJ, Mortier L (2019). Pembrolizumab versus ipilimumab in advanced melanoma (KEYNOTE-006): post-hoc 5-year results from an open-label, multicentre, randomised, controlled, phase 3 study. Lancet Oncol.

[3] Le DT, Uram JN, Wang H, Bartlett BR, Kemberling H, Eyring AD (2015). PD-1 Blockade in Tumors with Mismatch-Repair Deficiency. N Engl J Med.

[4] Le DT, Kim TW, Van Cutsem E, Geva R, Jäger D, Hara H (2020). Phase II Open-Label Study of Pembrolizumab in Treatment-Refractory, Microsatellite Instability-High/Mismatch Repair-Deficient Metastatic Colorectal Cancer: KEYNOTE-164. J Clin Oncol.

[5] Mondini M, Levy A, Meziani L, Milliat F, Deutsch E (2020). Radiotherapy-immunotherapy combinations - perspectives and challenges. Mol Oncol.

[6] Deutsch E, Chargari C, Galluzzi L, Kroemer G (2019). Optimising efficacy and reducing toxicity of anticancer radioimmunotherapy. Lancet Oncol.

[7] Demaria S, Guha C, Schoenfeld J, Morris Z, Monjazeb A, Sikora A (2021). Radiation dose and fraction in immunotherapy: one-size regimen does not fit all settings, so how does one choose?. J Immunother Cancer.

[8] Theelen WSME, Chen D, Verma V, Hobbs BP, Peulen HMU, Aerts JGJV (2021). Pembrolizumab with or without radiotherapy for metastatic non-small-cell lung cancer: a pooled analysis of two randomised trials. Lancet Respir Med.

[9] Spigel DR, Faivre-Finn C, Gray JE, Vicente D, Planchard D, Paz-Ares L (2022). Five-Year Survival Outcomes From the PACIFIC Trial: Durvalumab After Chemoradiotherapy in Stage III Non-Small-Cell Lung Cancer. J Clin Oncol.

[10] Zhu X, Cao Y, Liu W, Ju X, Zhao X, Jiang L, Ye Y, Jin G, Zhang H (2022). Stereotactic body radiotherapy plus pembrolizumab and trametinib versus stereotactic body radiotherapy plus gemcitabine for locally recurrent pancreatic cancer after surgical resection: an open-label, randomised, controlled, phase 2 trial. Lancet Oncol.

[11] Altorki NK, McGraw TE, Borczuk AC, Saxena A, Port JL, Stiles BM (2021). Neoadjuvant durvalumab with or without stereotactic body radiotherapy in patients with early-stage non-small-cell lung cancer: a single-centre, randomised phase 2 trial. Lancet Oncol.

[12] Abravan A, Faivre-Finn C, Kennedy J, McWilliam A, van Herk M (2020). Radiotherapy-Related Lymphopenia Affects Overall Survival in Patients With Lung Cancer. J Thorac Oncol.

[13] Taguchi A, Furusawa A, Ito K, Nakajima Y, Shimizuguchi T, Hara K, Takao M, Kashiyama T, Kino N, Karasawa K, Yasugi T (2020). Postradiotherapy persistent lymphopenia as a poor prognostic factor in patients with cervical cancer receiving radiotherapy: a single-center, retrospective study. Int J Clin Oncol.

[14] Newman AM, Liu CL, Green MR, Gentles AJ, Feng W, Xu Y, Hoang CD, Diehn M, Alizadeh AA (2015). Robust enumeration of cell subsets from tissue expression profiles. Nat Methods.

[15] Steen CB, Liu CL, Alizadeh AA, Newman AM (2020). Profiling Cell Type Abundance and Expression in Bulk Tissues with CIBERSORTx. Methods Mol Biol.

[16] Chen EX, Jonker DJ, Loree JM, Kennecke HF, Berry SR, Couture F (2020). Effect of Combined Immune Checkpoint Inhibition vs Best Supportive Care Alone in Patients With Advanced Colorectal Cancer: The Canadian Cancer Trials Group CO.26 Study. JAMA Oncol.

[17] Newey A, Griffiths B, Michaux J, Pak HS, Stevenson BJ (2019). Immunopeptidomics of colorectal cancer organoids reveals a sparse HLA class I neoantigen landscape and no increase in neoantigens with interferon or MEK-inhibitor treatment. J Immunother Cancer.

[18] Wang C, Sandhu J, Ouyang C, Ye J, Lee PP, Fakih M (2021). Clinical Response to Immunotherapy Targeting Programmed Cell Death Receptor 1/Programmed Cell Death Ligand 1 in Patients With Treatment-Resistant Microsatellite Stable Colorectal Cancer With and Without Liver Metastases. JAMA Netw Open.

[19] Fakih M, Raghav KPS, Chang DZ, Larson T, Cohn AL, Huyck TK (2023). Regorafenib plus nivolumab in patients with mismatch repair-proficient/microsatellite stable metastatic colorectal cancer: a single-arm, open-label, multicentre phase 2 study. EClinicalMedicine.

[20] Lee JC, Mehdizadeh S, Smith J, Young A, Mufazalov IA, Mowery CT (2020). Regulatory T cell control of systemic immunity and immunotherapy response in liver metastasis. Sci Immunol.

[21] Yu J, Green MD, Li S, Sun Y, Journey SN, Choi JE, et al. Liver metastasis restrains immunotherapy efficacy via macrophage-mediated T cell elimination. Nat Med. 2021;27:152–164. 10.1038/s41591-020-1131-x PMC8095049 33398162

[22] Segal NH, Cercek A, Ku G, Wu AJ, Rimner A, Khalil DN (2021). Phase II Single-arm Study of Durvalumab and Tremelimumab with Concurrent Radiotherapy in Patients with Mismatch Repair-proficient Metastatic Colorectal Cancer. Clin Cancer Res.

[23] Luke JJ, Onderdonk BE, Bhave SR, Karrison T, Lemons JM, Chang P (2020). Improved Survival Associated with Local Tumor Response Following Multisite Radiotherapy and Pembrolizumab: Secondary Analysis of a Phase I Trial. Clin Cancer Res.

[24] Hamon P, Gerbé De Thoré M, Classe M, Signolle N, Liu W, et al. TGFβ receptor inhibition unleashes interferon-β production by tumor-associated macrophages and enhances radiotherapy efficacy. J Immunother Cancer. 2022;10:e003519. 10.1136/jitc-2021-003519 PMC8932273 35301235

[25] Yamazaki T, Gunderson AJ, Gilchrist M, Whiteford M, Kiely MX, Hayman A (2022). Galunisertib plus neoadjuvant chemoradiotherapy in patients with locally advanced rectal cancer: a single-arm, phase 2 trial. Lancet Oncol.

[26] Morris VK, Overman MJ, Lam M, Parseghian CM, Johnson B (2022). Bintrafusp alfa, an anti-PD-L1:TGF-β trap fusion protein, in patients with ctDNA-positive, liver-limited metastatic colorectal cancer. Cancer Res Commun.

[27] Schoenfeld JD, Giobbie-Hurder A, Ranasinghe S, Kao KZ, Lako A, Tsuji J (2022). Durvalumab plus tremelimumab alone or in combination with low-dose or hypofractionated radiotherapy in metastatic non-small-cell lung cancer refractory to previous PD(L)-1 therapy: an open-label, multicentre, randomised, phase 2 trial. Lancet Oncol.

[28] Kim S, Wuthrick E, Blakaj D, Eroglu Z, Verschraegen C, Thapa R (2022). Combined nivolumab and ipilimumab with or without stereotactic body radiation therapy for advanced Merkel cell carcinoma: a randomised, open label, phase 2 trial. Lancet.

[29] Moretto R, Rossini D, Catteau A, Antoniotti C, Giordano M, Boccaccino A (2023). Dissecting tumor lymphocyte infiltration to predict benefit from immune-checkpoint inhibitors in metastatic colorectal cancer: lessons from the AtezoT RIBE study. J Immunother Cancer.

[30] Spurr LF, Martinez CA, Kang W, Chen M, Zha Y, Hseu R (2022). Highly aneuploid non-small cell lung cancer shows enhanced responsiveness to concurrent radiation and immune checkpoint blockade. Nat Cancer.

[31] Parikh AR, Szabolcs A, Allen JN, Clark JW, Wo JY, Raabe M (2021). Radiation therapy enhances immunotherapy response in microsatellite stable colorectal and pancreatic adenocarcinoma in a phase II trial. Nat Cancer.

[32] Sun R, Henry T, Laville A (2022). Imaging approaches and radiomics: toward a new era of ultraprecision radioimmunotherapy?. J Immunother Cancer.

[33] Darragh LB, Knitz MM, Hu J, Clambey ET, Backus J, Dumit A (2022). A phase I/Ib trial and biological correlate analysis of neoadjuvant SBRT with single-dose durvalumab in HPV-unrelated locally advanced HNSCC. Nat Cancer.

