# Supplementary material for: An international phase II trial and immune profiling of SBRT and atezolizumab in advanced pretreated colorectal cancer
Source: Mol Cancer. 2024 Mar 23;23:61. doi: 10.1186/s12943-024-01970-8 (PMC10960440; doi:10.1186/s12943-024-01970-8)
Supplement: Supplementary file 1 — Supplementary Material 1. [file 12943_2024_1970_MOESM1_ESM.docx]

**Figure S1 - Therapeutic schedule**

**
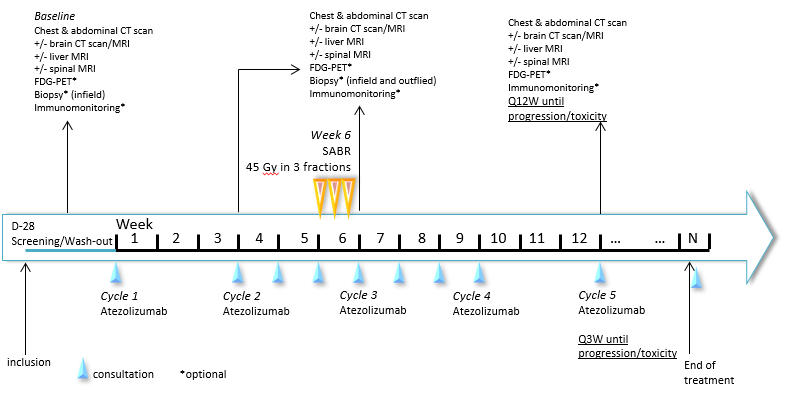
**

| **Figure S2. PFS and OS of according to MMR status** |
| --- |

**Table S1: Distribution of worst severity grade of adverse events over the whole treatment duration, regardless of causality**

|  | | | | Grade | | | | |
| --- | --- | --- | --- | --- | --- | --- | --- | --- |
| **SOC** | Preferred term |  | | 1 | 2 | 3 | 4 | 5 |
| **Blood and lymphatic system disorders** | Anemia | . | . | 1 (2%) | 7 (12%) | 5 (8%) |  |  |
|  | Coagulation Factor V decreased | . | . |  |  | 1 (2%) |  |  |
|  | Lymphopenia | . | . |  | 1 (2%) | 1 (2%) |  |  |
|  | Thrombopenia | . | . |  |  |  | 1 (2%) |  |
| **Cardiac disorder** | Atrial fibrillation | . | . |  | 1 (2%) |  |  |  |
| **Ear and labyrinth disorders** | Dysphonia | . | . | 1 (2%) |  |  |  |  |
|  | Hearing loss | . | . | 1 (2%) |  |  |  |  |
|  | Vertigo | . | . | 1 (2%) |  |  |  |  |
| **Endocrine disorders** | Serum Amylase increased | . | . | 1 (2%) |  |  |  |  |
| **Eye disorders** | Blurred vision | . | . | 1 (2%) |  |  |  |  |
| **Gastrointestinal disorders** | Abdominal Infection | . | . |  | 1 (2%) |  |  |  |
|  | Abdominal pain | . | . | 5 (8%) | 1 (2%) | 3 (5%) |  |  |
|  | Ascites | . | . |  | 1 (2%) |  |  |  |
|  | Biliary Stenosis | . | . |  |  | 1 (2%) |  |  |
|  | Bloating | . | . | 1 (2%) |  |  |  |  |
|  | Colonic obstruction | . | . |  |  | 1 (2%) |  | 1 (2%) |
|  | Constipation | . | . | 9 (15%) | 4 (7%) |  |  |  |
|  | Diarrhea | . | . |  | 2 (3%) |  |  |  |
|  | Dry mouth | . | . | 2 (3%) |  |  |  |  |
|  | Dyspepsia | . | . | 1 (2%) |  |  |  |  |
|  | Dysphagia | . | . | 1 (2%) |  |  |  |  |
|  | Gastric hemorrhage | . | . |  | 1 (2%) |  |  |  |
|  | Ileal obstruction | . | . |  |  | 1 (2%) |  |  |
|  | Lower gastrointestinal hemorrhage | . | . | 1 (2%) |  |  |  |  |
|  | Mucositis oral | . | . | 2 (3%) |  |  |  |  |
|  | Proctitis | . | . | 1 (2%) | 1 (2%) |  |  |  |
|  | Rectal hemorrhage | . | . |  | 1 (2%) |  |  |  |
|  | Small intestinal obstruction | . | . |  | 1 (2%) |  |  |  |
|  | Stomach pain | . | . | 1 (2%) |  |  |  |  |
| **General disorders and administration site conditions** | Asthenia | . | . | 1 (2%) | 1 (2%) | 1 (2%) |  | 1 (2%) |
|  | Chills | . | . | 1 (2%) |  |  |  |  |
|  | Edema limbs | . | . | 4 (7%) | 2 (3%) |  | 1 (2%) |  |
|  | Hyperhidrosis | . | . | 1 (2%) |  |  |  |  |
|  | Hypothermia | . | . | 1 (2%) | 1 (2%) |  |  |  |
|  | Non-cardiac chest pain | . | . | 1 (2%) |  |  |  |  |
|  | Pain | . | . | 7 (12%) | 2 (3%) | 1 (2%) |  |  |
| **Hepatobiliary disorders** | Alkaline Phosphatase increased | . | . |  | 1 (2%) |  |  |  |
|  | Hepatic Cytolysis | . | . |  |  | 2 (3%) |  |  |
|  | Hepatic failure | . | . |  |  |  |  | 1 (2%) |
|  | Hepatic pain | . | . | 3 (5%) | 1 (2%) |  |  |  |
|  | LDH increased | . | . |  | 1 (2%) |  |  |  |
|  | CRP increased | . | . |  |  |  | 1 (2%) |  |
| **Immune system disorders** | Autoimmune Hepatitis | . | . |  |  | 1 (2%) |  |  |
| **Infections and infestations** | Mucosal infection | . | . | 1 (2%) |  |  |  |  |
|  | Mycosis | . | . | 1 (2%) |  |  |  |  |
|  | Pneumonitis | . | . |  | 1 (2%) |  |  |  |
|  | Rhinitis | . | . | 1 (2%) |  |  |  |  |
|  | Sepsis | . | . |  |  |  | 2 (3%) |  |
|  | Sinusitis | . | . |  | 1 (2%) |  |  |  |
|  | Urinary tract infection | . | . |  |  | 1 (2%) |  |  |
| **Investigations** | Alkaline phosphatase increased | . | . |  | 4 (7%) | 7 (12%) |  |  |
|  | Aspartate aminotransferase increased | . | . | 1 (2%) |  |  |  |  |
|  | Blood bilirubin increased | . | . | 2 (3%) |  | 1 (2%) |  |  |
|  | CPK increased | . | . | 2 (3%) |  | 1 (2%) | 1 (2%) |  |
|  | Cardiac troponin I increased | . | . |  |  | 1 (2%) |  |  |
|  | Creatinine increased | . | . | 3 (5%) | 1 (2%) |  |  |  |
|  | Eosinophilia | . | . |  | 1 (2%) |  |  |  |
|  | GGT increased | . | . | 1 (2%) | 1 (2%) | 4 (7%) | 1 (2%) |  |
|  | Lipase increased | . | . |  |  | 3 (5%) |  |  |
|  | Lymphocyte count decreased | . | . |  |  | 4 (7%) |  |  |
|  | Platelet count decreased | . | . | 1 (2%) |  | 1 (2%) |  |  |
|  | Prothrombine decreased | . | . |  |  | 1 (2%) |  |  |
|  | Serum amylase increased | . | . |  |  | 1 (2%) |  |  |
|  | Weight loss | . | . | 2 (3%) | 1 (2%) |  |  |  |
| **Metabolism and nutrition disorders** | Anorexia | . | . | 2 (3%) |  |  |  |  |
|  | Hyperglycemia | . | . |  | 2 (3%) |  |  |  |
|  | Hypertriglyceridemia | . | . | 1 (2%) |  |  |  |  |
|  | Hypoalbuminemia | . | . |  | 6 (10%) |  |  |  |
|  | Hypoglycemia | . | . |  | 1 (2%) |  |  |  |
|  | Hypokalemia | . | . | 2 (3%) | 2 (3%) |  |  |  |
|  | Hypomagnesemia | . | . | 1 (2%) |  |  |  |  |
|  | Hypophosphatemia | . | . |  | 1 (2%) | 1 (2%) |  |  |
|  | Hypophosphoremia | . | . |  |  | 1 (2%) |  |  |
|  | Hypothermia | . | . | 1 (2%) |  |  |  |  |
| **Musculoskeletal and connective tissue disorders** | Arthralgia | . | . | 3 (5%) |  |  |  |  |
|  | Back pain | . | . | 3 (5%) |  |  |  |  |
|  | Bone pain | . | . | 2 (3%) |  |  |  |  |
|  | Fracture | . | . |  |  |  | 1 (2%) |  |
|  | Myalgia | . | . | 3 (5%) |  |  |  |  |
|  | Myositis | . | . |  |  | 1 (2%) |  |  |
|  | Pain in extremity | . | . |  | 2 (3%) |  |  |  |
|  | Spinal fracture | . | . |  |  | 1 (2%) |  |  |
| **Neoplasms benign, malignant and unspecified** | Brain Metastases | . | . |  |  |  | 1 (2%) |  |
|  | Tumor pain | . | . | 10 (17%) | 8 (14%) |  |  |  |
| **Nervous system disorders** | Asthenia | . | . |  |  | 1 (2%) |  |  |
|  | Headache | . | . | 2 (3%) |  |  |  |  |
|  | Nervous system disorders - Other | . | . | 1 (2%) |  |  |  |  |
|  | Neuralgia | . | . | 1 (2%) |  |  |  |  |
|  | Paresthesia | . | . | 1 (2%) |  |  |  |  |
|  | Peripheral neuropathy | . | . | 1 (2%) |  |  |  |  |
|  | Peripheral motor neuropathy | . | . | 4 (7%) |  |  |  |  |
|  | Peripheral sensory neuropathy | . | . | 1 (2%) | 1 (2%) |  |  |  |
| **Psychiatric disorders** | Depression | . | . | 3 (5%) | 1 (2%) |  |  |  |
| **Renal and urinary disorders** | Urinary Tract infection | . | . | 1 (2%) |  |  |  |  |
|  | Urinary retention | . | . | 1 (2%) |  |  |  |  |
|  | Urinary tract obstruction | . | . |  |  | 1 (2%) |  |  |
| **Reproductive system and breast disorders** | Prostate hypertrophy | . | . | 1 (2%) |  |  |  |  |
|  | Uterine hemmorage | . | . | 2 (3%) |  |  |  |  |
| **Respiratory, thoracic and mediastinal disorders** | Allergic rhinitis | . | . |  | 1 (2%) |  |  |  |
|  | Cough | . | . | 1 (2%) |  |  |  |  |
|  | Dyspnea | . | . | 1 (2%) |  |  |  |  |
|  | Pleural effusion | . | . |  | 2 (3%) |  |  |  |
|  | Pneumonitis | . | . |  | 1 (2%) |  |  |  |
|  | Pneumothorax | . | . |  |  | 1 (2%) |  |  |
| **Skin and subcutaneous tissue disorders** | Dry skin | . | . | 2 (3%) |  |  |  |  |
|  | Folliculitis | . | . | 1 (2%) |  |  |  |  |
|  | Psoriasis | . | . | 1 (2%) |  |  |  |  |
|  | Skin hyperpigmentation | . | . | 1 (2%) |  |  |  |  |
|  | Skin infection | . | . |  |  | 1 (2%) |  |  |
| **Vascular disorders** | Hypertension | . | . | 2 (3%) |  |  |  |  |
|  | Hypotension | . | . |  | 1 (2%) |  |  |  |
|  | Pulmonary Embolism | . | . |  | 1 (2%) |  |  |  |

**Table S2: Tumor biopsy**

| **#inclusion** | **Group** | **MSS/MSI status** | **Metastasis localisation** | **Baseline** | **Week 3** | **Week 7** |
| --- | --- | --- | --- | --- | --- | --- |
| #1 | SD/PR/CR | MSI-h | retroperitoneum |  | X |  |
| #2 | SD/PR/CR | MSI-h | mesentery | X | X |  |
| #3 | SD/PR/CR (Elite) | MSI-h | liver | X | X | X |
| #9 | SD/PR/CR | MSS | liver | X | X | X |
| #14 | PD | MSS | lung | X | X |  |
| #20 | PD | MSS | lung | X |  |  |
| #22 | PD | MSS | liver | X | X |  |
| #26 | PD | MSS | liver | X | X | X |
| #30 | PD | MSS | liver | X | X | X (no FFPE) |
| #31 | PD | MSI-h | liver | X |  |  |
| #34 | SD/PR/CR | MSI-h | liver | X | X | X |
| #36 | SD/PR/CR (Elite) | MSI-h | supraclavicular node | X | X | X |

**Figure S3: Scatter dot plot showing the differences in absolute lymphocyte blood counts (A) and neutrophil to lymphocyte ratios (B) according to response to treatment and timepoint. Mean with standard deviation (SD) are represented. The red dotted line indicated the lymphopenia rate.**

**A**

**B**

**G**

**Figure S4: Comparison of tumor immune infiltration according to disease control rate (defined as lack of disease progression) in baseline biopsies by different IHC markers**
